# Research Progress towards Understanding the Unique Interfaces between Concentrated Electrolytes and Electrodes for Energy Storage Applications

**DOI:** 10.1002/advs.201700032

**Published:** 2017-03-31

**Authors:** Jianming Zheng, Joshua A. Lochala, Alexander Kwok, Zhiqun Daniel Deng, Jie Xiao

**Affiliations:** ^1^ Chemistry & Biochemistry Department University of Arkansas Fayetteville AR 72701 USA; ^2^ Energy and Environment Directorate Pacific Northwest National Laboratory 902 Battelle Boulevard Richland WA 99354 USA

**Keywords:** batteries, concentrated electrolytes, interfacial stability, solvation structures, solid electrolyte interphase (SEI)

## Abstract

The electrolyte is an indispensable component in all electrochemical energy storage and conversion devices with batteries being a prime example. While most research efforts have been pursued on the materials side, the progress for the electrolyte is slow due to the decomposition of salts and solvents at low potentials, not to mention their complicated interactions with the electrode materials. The general properties of bulk electrolytes such as ionic conductivity, viscosity, and stability all affect the cell performance. However, for a specific electrochemical cell in which the cathode, anode, and electrolyte are optimized, it is the interface between the solid electrode and the liquid electrolyte, generally referred to as the solid electrolyte interphase (SEI), that dictates the rate of ion flow in the system. The commonly used electrolyte is within the range of 1–1.2 m based on the prior optimization experience, leaving the high concentration region insufficiently recognized. Recently, electrolytes with increased concentration (>1.0 m) have received intensive attention due to quite a few interesting discoveries in cells containing concentrated electrolytes. The formation mechanism and the nature of the SEI layers derived from concentrated electrolytes could be fundamentally distinct from those of the traditional SEI and thus enable unusual functions that cannot be realized using regular electrolytes. In this article, we provide an overview on the recent progress of high concentration electrolytes in different battery chemistries. The experimentally observed phenomena and their underlying fundamental mechanisms are discussed. New insights and perspectives are proposed to inspire more revolutionary solutions to address the interfacial challenges.

## 1 Introduction

The ever‐increasing energy demand and global environmental concerns have accelerated the efforts to develop low‐emission or zero‐emission electric vehicles (EVs) powered by high energy batteries.^1^ There is also increasing demand for high‐energy‐density battery systems for stationary wind and solar energy storage. Rechargeable lithium‐ion batteries (LIBs) and lithium (Li) metal batteries are considered the significant power sources to meet these demands. Depending on the specific applications, various batteries should find their way to fit into different systems. For example, the prioritory concerns of LIBs for hybrid electric vehicles (HEVs) or pure EVs are their energy density and safety properties. For storing renewable energy, reliabilty and cost are more important.^2^ While many research interests have been focused on materials chemistry,^3^ and electrolytes,^4^ the understanding of their derived interfaces has made much less progress due to the complexity of electrolyte decomposition in dynamic conditions and on various substrates with different surface properties. However, interfaces do play a critically important role in determining the mass flow and electrochemical kinetics, and thus the power, stability, and safety of LIBs.^4^

The widely adopted non‐aqueous electrolyte for LIBs is lithium hexafluorophosphate (LiPF_6_) dissolved in mixtures of alkyl carbonates such as dimethyl carbonate (DMC), ethyl methyl carbonate (EMC), ethylene carbonate (EC) and propylene carbonate (PC).^5^ However, LiPF_6_ is thermodynamically unstable and is sensitive to moisture. Other popular salts include lithium bis(trifluoromethylsulfonyl)imide (LiTFSI, or LiTFSA) and lithium bis(fluorosulfonyl)imide (LiFSI, or LiFSA), which are more stable against moisture and have attracted many interests in battery research. However, LiTFSI and LiFSI have corrosion issues with the aluminum (Al) current collector at voltages above ca. 3.7 V.^6^ Therefore, the electrochemical window is restricted in the electrolytes containing LiTFSI/LiFSI as the salt. The selection of solvents depend on the specific applications and the operating environments of the batteries. For example, electrolyte based on PC has higher ionic conductivity at low temperatures owing to the lower melting point (−48.8 °C) of PC than other solvents such as EMC (−14.5 °C), and EC (36.4 °C).^5^ EC is considered the magic ingrediant to passivate the layered structure of graphite by forming a protective solid electrolyte interphase (SEI) layer on graphite surface.^7^ Ethers are more compatible with radicals so dioxolane (DOL)/dimethoxyethane (DME) is generally used in Li‐S or Li‐O_2_ batteries.^8^

**Figure 1:**
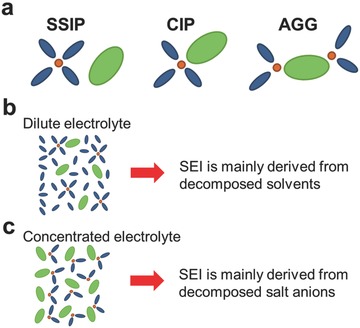
(a) Representative Li^+^ cation solvate species (SSIP, CIP and AGGs) in dilute and concentrated electrolytes. Schematic illustration of the electrolyte reduction mechanism at the electrode/electrolyte interface in (b) dilute and (c) concentrated electrolytes.

The synergistic effects of both salt and solvent molecules affect the quality of the SEI derived from the electrolytes. From a solvation structure point of view, a lithium ion (Li^+^) is normally coordinated with 3 to 4 solvent molecules in the dilute electrolyte solution, which is dominated with solvent‐separated ion pairs (SSIPs) and free solvent molecules (**Figure** **1**a).^9^ Therefore, the SEI layer formed in regular electrolytes is mainly derived by the decomposition of electrolyte solvents (Figure 1b). In the case of concentrated electrolyte (typically > 3.0 m, m being molarity (mol L^–1^)), the coordination number is reduced to 1−2 due to the scarcity of solvent molecules. Salt anions enter the solvation sheath to form contact ion pairs (CIPs) and cation‐anion aggregates (AGGs) (Figure 1a). These salt anions thus participate in the SEI layer formation by shifting from a solvent decomposition to a salt anion decomposition/reaction as a result of the increase of Li salt concentration (Figure 1c).^10^

The pros and cons of dilute and concentrated electrolyte systems are briefly compared in **Table** **1**. In concentrated electrolyte, the SEI layer is mainly derived from anions and is typically rich in LiF as currently reported.^11^ The LiF‐enhanced SEI has improved qualities such as better adhesion to the electrode surface and is a thinner but much denser protective layer with enhanced mechanical property.^12^ The salt concentrating strategy also relieves safety concerns due to the enhanced thermal stability as well as the reduced flammability of the high concentration electrolyte.^13^ Despite their relatively high viscosity, some unique functionalities of concentrated electrolytes have been discovered, e.g., the reversible Li^+^ ion intercalation and de‐intercalation with graphite in EC‐free electrolytes,[[qv: 12a]] and suppresion of Al corrosion in LiPF_6_‐free electrolytes during high voltage operation.^10^ In addition, these electrolytes are reported to protect the Li metal anode,^14^ and enhance the cycling stability of high energy Li metal based batteries, i.e. Li‐S batteries,^15^ organic Li batteries,^16^ and Li‐O_2_ batteries.^17^ Moreover, highly concentrated electrolytes could largely improve the energy density of “dual‐carbon” batteries operating with PF_6_^−^ anion intercalation into carbon cathodes and Li^+^ cation intercalation into graphite anodes.^18^ In addition to non‐aqueous Li based batteries, the positive effects of the salt concentrating strategy has also been observed in sodium ion (Na^+^) batteries, magnesium ion (Mg^2+^) batteries,^19^ and aqueous LIBs.^20^ However, the formation, evolution, and the nature of the SEI derived from concentrated electrolytes are not conclusively known yet. How the concentrated anions affect the original electrical double layer and the subsequent formation of the SEI is arguable.

**Table 1:** Comparison of dilute and concentrated electrolyte systems

Physicochemical Property	Dilute Electrolyte	Concentrated Electrolyte
Representative components in the bulk electrolyte	Solvent‐separated ion pairs (SSIP) and free solvent molecules	Contact ion pairs (CIPs) and cation‐anion aggregates (AGGs)
Representative components of the SEI	A combination of inorganic salts decomposed from the solute and organic species derived from solvents	Dominated by inorganic species from decomposed anions, or from partially precipitated solutes
Flammability	High	Low
Thermal Stability	Poor	Good
Reductive Stability	Low	High
Oxidative Stability	Low	High
Viscosity	Low	High
Ionic Conductivity	10^–2^ S cm^–1^	10^–3^–10^–2^ S cm^–1^
Wettability	Good	Relatively poor
Electrode reaction kinetics	Slow	Fast
Power density	High	May exceed conventional LiPF_6_/EC‐based electrolyte at certain circumstances
Energy density	High	High
Cost	Low	High

In an earlier review, Yamada et al. disscussed the superconcentrated electrolyte for advanced lithium battery applications with a focus on the solution structure and physicochemical properties of concentrated electrolytes.^21^ In this article, recent advances of concentrated electrolytes in various battery systems will be reviewed first, including conventional Li‐ion batteries, ‘beyond Li‐ion’ energy storage systems, and aqueous‐based energy storage systems. Different SEI formation mechanisms in concentrated electrolytes will then be compared and discussed. Finally, new insights and perspectives will be proposed which may inspire more revolutionary solutions to address the interfacial challenges in energy storage research.

## 2 Discussion

### 2.1 Concentrated Electrolyte for Li‐ion Battery

#### 2.1.1 EC‐Free Concentrated Electrolyte for Graphite Anode

The successful development of EC‐based electrolytes is one of the key milestones in the commercialization of LIBs. EC is generally accepted as a necessary component in a LIB electrolyte because of its capability in building up a protective layer, consisting of organic polymeric species and other inorganic components such as Li_2_CO_3_, Li_2_O, and LiF, on the graphite surface that prevent the solvent co‐intercalation.^22^ However, the high viscosity of EC affects the low‐temperature cell performance and its fast charging performance.^23^ Other solvents need to be mixed with EC to balance the overall properties of SEI layers formed. Early efforts to intercalate Li^+^ ion into a graphite lattice failed in electrolytes containing solvents such as PC, dimethyl sulfoxide (DMSO), acetonitrile (AN), and sulfolane (SL). For instance, PC‐based electrolytes have attracted intense research interests owing to their higher ionic conducitivity at lower temperatures compared to EC‐based electrolytes. However, in a typical 1 m electrolyte solution, PC cannot form a protective SEI on the graphite surface and continuously co‐intercalates into the graphite with the solvated Li^+^ ion, leading to the exfoliation of graphite and consequently to cell failure.[[qv: 7a]]

**Figure 2:**
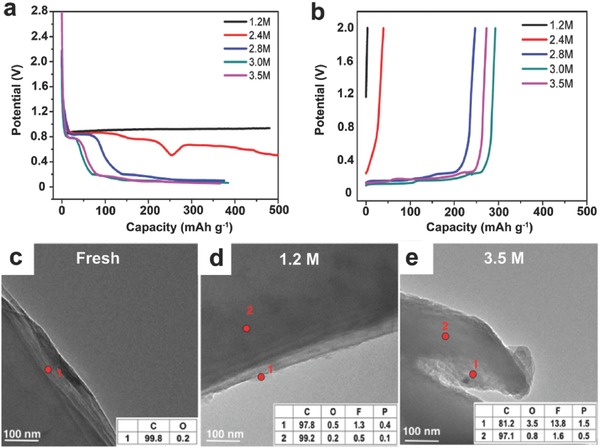
(a) Li intercalation profiles and (b) Li de‐intercalation profiles for BF‐graphite/Li cells cycled with various concentrations of LiPF_6_/PC electrolytes. TEM images of fresh and cycled graphite anodes with different concentrations of LiPF_6_/PC electrolytes: (c) Fresh graphite, (d) 1.2 m, and (e) 3.5 m. The inset presents the element composition detected by energy‐dispersive X‐ray spectroscopy (EDX). Reproduced with permission.[[qv: 11a]] Copyright 2013 American Chemical Society.

Recent studies have testified that increasing the Li salt concentration could enable the graphite electrode reactions in organic solvents other than EC, which is summarized in **Table** **2**. Jeong et al. demonstrated the successful intercalation of Li^+^ ions into graphite to form a stage I Li‐graphite intercalation compound (Li−GIC) in a concentrated electrolyte with pure PC as a solvent, e.g. 2.72 m lithium bis(perfluoroethylsulfonyl)imide [LiN(SO_2_C_2_F_5_)_2_, LiBETI]/PC.^24^ In a separate publication, they further investigated the effects of electrolyte concentration on the interfacial reactions between graphite and PC‐based electrolytes during the charge and discharge processes.^25^ A very thin film (thickness of ≈8 nm) formed on the graphite surface effectively suppressed both the co‐intercalation of PC molecules and the further electrolyte decomposition on the graphite surface. However, the detailed mechanism of forming the different surface films in relation to the electrolyte concentration was not clear at that time. Later, Nie et al. correlated the SEI formation on graphite with the solution structures of different concentrations of LiPF_6_/PC electrolytes.[[qv: 11a]] Varying the concentration of LiPF_6_ largely changes the solution structure, which consequently alters the predominant mechanism of electrolyte reduction at the electrode interface (Figure 1b,c). At a low concentration of LiPF_6_ in PC (1.2 m), the solution structure is dominated by SSIP (Li^+^(PC)_4_//PF_6_^−^), with the primary reduction product of the electrolyte being lithium propylene dicarbonate (LPDC). This loose surface film could not prevent the sustained electrolyte reduction and no lithiation of the graphite (**Figure** **2**a–d) occured at all. An SEI layer dominated with carbonate components is also considered thermodynamically unstable.^26^ At high concentrations of LiPF_6_ in PC (3.0−3.5 m), the solution structure is dominated by CIPs (Li^+^(PC)_3_PF_6_^−^), with the primary reduction product of the electrolyte being LiF (Figure 2e). This thin and compact SEI layer, enriched by LiF and thermodynamically more stable, adheres well to the graphite surface and inhibits the unwanted electrolyte reductions, thereby enabling the reversible lithiation and delithiation of the graphite electrodes.

**Table 2:** The EC‐free superconcentrated electrolytes that could enable the successful intercalation of lithium ions into the graphite electrodes. The concentration listed in this table is in molarity (mol L^–1^)

Solvent	Lithium Salt	Successful Concentration	Reference
PC	LiBETI [LiN(SO_2_C_2_F_5_)_2_]	2.72 m	Jeong et al.^24^
PC	LiPF_6_	3.0 m	Nie et al.[[qv: 11a]]
PC	LiPF_6_	3.0 m	Ding et al.^82^
PC	LiClO_4_	3.8 m	Kim et al.^83^
DME	LiFSI (LiN(SO_2_F)_2_)	3.6 m	Yamada et al.^84^
AN	LiTFSI (LiN(SO_2_CF_3_)_2_)	4.5 m	Yamada et al.[[qv: 11b]]
DMSO	LiTFSI (LiN(SO_2_CF_3_)_2_)	3.2 m	Yamada et al.[[qv: 12a]]
SL	LiTFSI (LiN(SO_2_CF_3_)_2_)	3.0 m	Yamada et al.[[qv: 12a]]
THF	LiTFSI (LiN(SO_2_CF_3_)_2_)	3.0 m	Yamada et al.[[qv: 12a]]
EA	LiPF_6_ + LiFSI	0.5 m LiPF_6_ + 5 m LiFSI	Petibon et al.^85^

**Figure 3:**
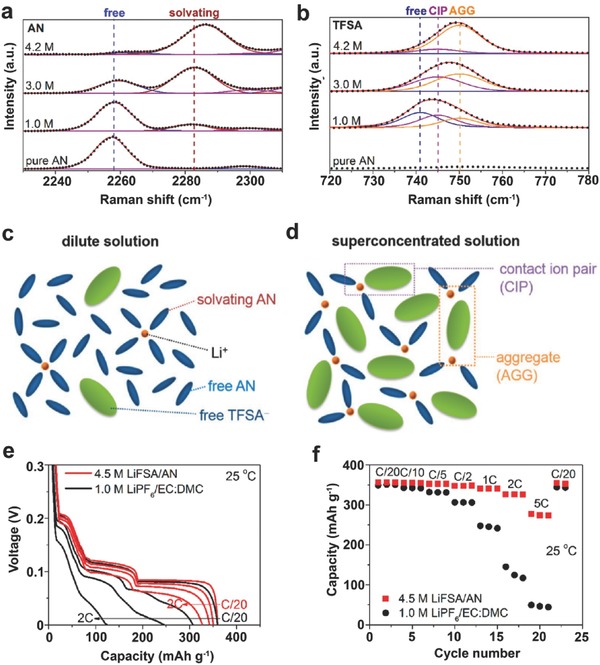
Raman spectra of LiTFSI/AN solutions in (a) 2230−2310 cm^−1^ (C≡N stretching mode of AN) and (b) 720−780 cm^−1^ (S−N stretching, C−S stretching, and CF_3_ bending mode of TFSI^−^). (c, d) Representative environments of Li^+^ ions in (c) a dilute solution and (d) a superconcentrated solution. (e) Li^+^ ion intercalation voltage curves of a Li||graphite half cell with superconcentrated 4.5 m LiFSI/AN and commercial 1.0 m LiPF_6_/EC‐DMC electrolytes at various C‐rates at 25 °C. (f) Rate performance of graphite electrode in the two electrolytes at 25 °C. Reproduced with permission.[[qv: 11b]] Copyright 2014 American Chemical Society.

Recently, Yamada et al. have further proposed to apply the salt‐concentrating strategy to the graphite intercalations in various other organic solvents that are usually considered incapable to produce effective SEI layers on graphite surfaces, including AN, DMSO, DME, THF, and SL (as listed in Table 2).[[qv: 12a]] For example, AN could be a promising solvent for LIBs considering its good stability against oxidation at high voltage vs. Li/Li^+^. However, it does not find many applications in batteries because of its crucially poor stability against reduction at voltages as high as ca. 1.6 V.^27^ By applying a superconcentrated LiTFSI (>4 m) in AN solution as a battery electrolyte, Yamada et al. demonstrated the reversible Li^+^ ion intercalation into a graphite electrode.[[qv: 11b]] Based on their Raman spectroscopy analysis (**Figure** **3**a,b), with an increase of LiTFSI concentration from 1.0 m to 4.2 m, the solvation structure around Li^+^ ion decreased from 3‐ or 4‐fold AN coordination to a 2‐fold AN coordination on average (Figure 3c,d). With the increase of Li‐salt concentration, the free TFSI^−^ anions diminished to form CIPs and AGGs over 3.0 m. At 4.2 m, nearly all the TFSI^−^ anions existed as AGGs with strong coulombic interactions with multiple Li^+^ cations. The salt‐superconcentrated solution is featured by a fluidic polymeric network of mutually interacting TFSI^−^ anions and Li^+^ cations in the presence of two AN molecules solvating each Li^+^, which modifies the forming mechanism of the SEI layer and provides unusual reductive stability. In this case, TFSI^−^ anions are preferentially reduced to form a TFSI‐derived surface film on the graphite surface, which is the origin of the improved reductive stability to allow for reversible Li^+^ ion intercalation into the graphite electrode. The improved charging rate capability of the graphite electrode in superconcentrated LiTFSI/AN electrolyte further supports the conclusion that SEI layers derived from the concentrated electrolytes have very unique properties to allow for prompt Li^+^ ion transfer (Figure 3e,f).

#### 2.1.2 Concentrated Electrolyte for Suppressing Al Corrosion

**Figure 4:**
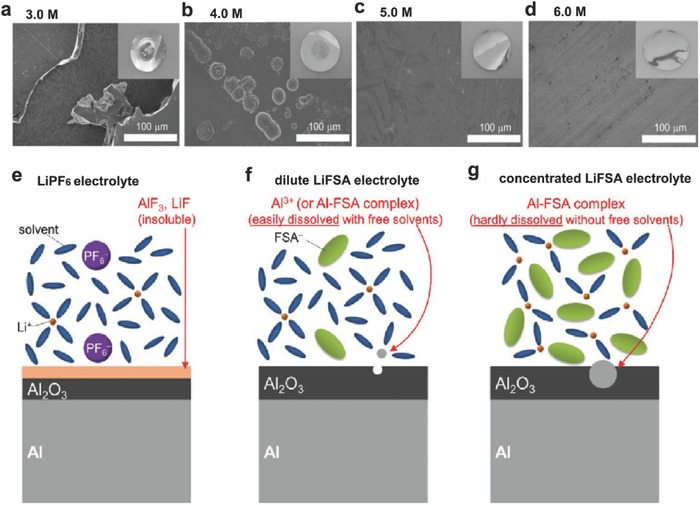
(a–d) SEM images of the Al electrode surface kept at 4.5 V for 10 h in a) 3.0 m, b) 4.0 m, c) 5.0 m, and d) 6.0 m LiFSI/AN electrolytes. Photographed images are shown as insets. Schematic illustrations of the behavior of Al electrodes in e) conventional LiPF_6_‐based electrolyte, f) dilute LiFSI/AN electrolyte with a considerable amount of free solvent molecules, and g) highly concentrated LiFSI/AN electrolyte without free solvent molecules. Reproduced with permission.[[qv: 29b]]

LiFSI and LiTFSI have received increasing attention as alternative Li salts for LIBs due to their higher chemical and thermal stability relative to LiPF_6_.^28^ Of note, impurities and residual moistures in LiFSI affect its thermal stability. High purity and high quality LiFSI salt is indispensable to achieve consistent thermal stability and electrochemical results. Although LiFSI is not as stable as LiTFSI, it has demonstrated a better thermal stability than LiPF_6_.[[qv: 28a]] The challenge associated with the use of LiFSI/LiTFSI salts is the severe corrosion of the Al current collector in regular concentration electrolyte solution during charge to high voltages.^29^ Recently, high concentrations of LiFSI/LiTFSI salt have been adopted to improve the electrolyte compatibility with the Al current collector.[[qv: 10,13,29b,30]] Yamada et al. studied the corrosion prevention mechanism of Al in highly concentrated LiFSI/AN electrolytes from the solution structure point of view.[[qv: 29b]] In their strudy, the oxidative corrosion of Al is effectively suppressed up to 4.5 V (vs. Li/Li^+^) when the salt concentration is over 5 m (**Figure** **4**a–d). In conventional LiPF_6_‐based electrolytes, the thermal and electrochemical decomposition or hydrolysis of PF_6_^−^ anion produces the F^−^ anions, which is a strong Lewis base that strongly binds to the Al^3+^ generated at high voltages, thus effectively stabilizing the Al metal with the formation of LiF/AlF_3_ passivation layer (Figure 4e).^31^ For LiFSI‐based electrolyte, the presence of the F^−^ anions is limited due to the superior thermal and chemical stability of the FSI^−^ anion. In a dilute LiFSI/AN electrolyte, the Al^3+^ formed at high voltage is solvated by free AN solvent and could easily diffuse from the Al surface to bulk electrolyte, leading to the continous corrosion of Al (Figure 4f). In the highly concentrated electrolyte, Al corrosion was, however, significantly suppressed due to the following two aspects (Figure 4g): i) declined solvation of Al^3+^ due to lack of free solvent molecules and ii) reduced diffusivity of Al(FSI)_3_ complex in concentrated electrolyte. Benifiting from the stabilized interface, a 4V‐class Li||LiMn_2_O_4_ cell using concentrated LiFSI/AN electrolyte demonstrated a decent reversible cycling with high coulombic efficiency (CE) at a low C/10 rate. Matsumto et al. reported the suppression of Al corrosion using high concentration LiTFSI/EC‐DEC electrolyte.^30^ They ascribed this suppression mechanism to the shortened distance between the TFSI^−^ anion and the Li^+^ cation, which facilitates to form a stabilized LiF‐rich SEI layer. Henderson et al. suggested that TFSI^−^ anions with strong C–F bonds were too stable to be oxidized to form F^−^ anions.^13^ Therefore, the Al current collector cannot be passiviated in a diluted LiTFSI/EC electrolyte.[[qv: 29b]] Instead, for the concentrated LiTFSI/solvent electrolytes, the absence of free solvent molecules along with the extensive coordination of the electron lone pairs on both solvent and anions to the positively‐charged Li^+^ cations improves the anodic stability of the Al surface. The solubility of Al(TFSI)_3_ complexes is expected to be much lower in concentrated electrolyte, because the solvent molecules and anions are extensively coordinated. The high concentration of TFSI^−^ anions at the electrode/electrolyte interface serves as a barrier hindering the access of solvent molecules to the electrode, further preventing Al dissolution from the electrode surface.

**Figure 5:**
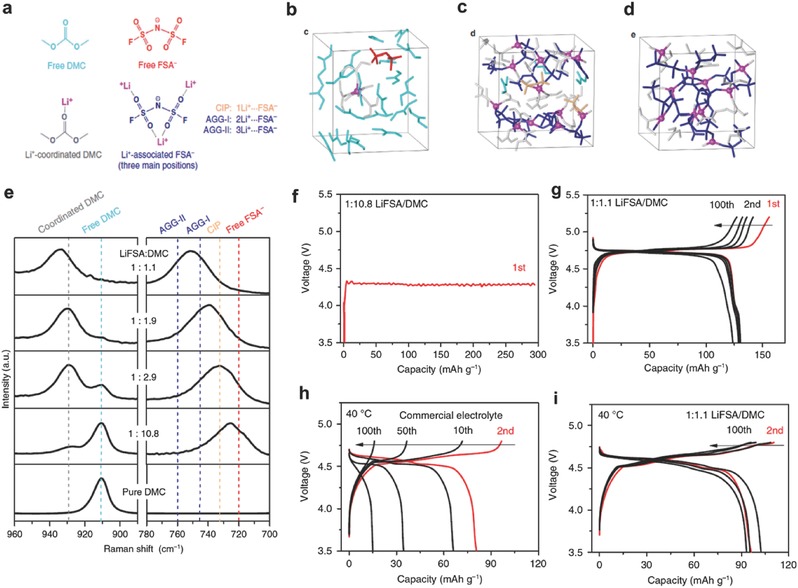
(a) The main species in the LiFSI/DMC solutions. (b–d) Snapshots of typical equilibrium trajectories obtained by DFT‐MD simulations: (b) dilute solution (<1 m), (c) moderately concentrated solution (ca. 4 m), and (d) superconcentrated solution (ca. 5.5 m). Free and coordinated DMC molecules are marked in light blue and grey, respectively. Free, CIP and AGG states of FSI^−^ anions are marked in red, orange and dark blue, respectively. (e) Raman spectra of LiFSI/DMC solutions with various salt‐to‐solvent molar ratios. Voltage profiles of Li||LiNi_0.5_Mn_1.5_O_4_ half‐cells using (f) dilute 1:10.8 and (g) superconcentrated 1:1.1 LiFSI/DMC electrolytes at C/5 and RT. Voltage profiles of graphite||LiNi_0.5_Mn_1.5_O_4_ full cells using (h) a 1.0 m LiPF_6_/EC‐DMC electrolyte and (i) a superconcentrated 1:1.1 LiFSI/DMC electrolyte at C/5 and 40 °C. Reproduced with permission.^10^ Copyright 2016 Nature Publishing Group.

An electrolyte with high oxidation stability is critical for the development of next‐generation 5V‐class LIBs using high voltage spinel LiNi_0.5_Mn_1.5_O_4_ cathodes or Li‐ and Mn‐rich cathodes to further increase the energy density of LIBs.^32^ The LiPF_6_ salt in conventional electrolyte is chemically unstable, and the resulting HF acidic species accelerates the dissolution of transition metal (TM) ions from the active material. Replacing LiPF_6_ with stable Li salts such as LiFSI and LiTFSI mitigates TM ion dissolution, but severe Al corrosion occurs instead. The previously mentioned concentrated imide‐based electrolytes showed mitigated anodic Al dissolution, but the stable operating voltage is still limited to 4.3–4.5 V.[[qv: 29b]] Wang et al. reported a new electrolyte design by mixing LiFSI with DMC solvent at extremely high salt concentrations (**Figure** **5**a).^10^ They obtained an unusual liquid that showed a three‐dimensional (3D) network of anions and solvent molecules that strongly coordinate to Li^+^ ions (Figure 5b–d), featured with AGG clusters as the predominant solvate species, rare amount of CIPs, and the absence of free solvent molecules (Figure 5e). This superconcentrated LiFSI/DMC electrolyte effectively inhibited the dissolution of both Al and TM ions up to 5 V, and enabled a sustainable operation of LiNi_0.5_Mn_1.5_O_4_ in Li half cells and graphite||LiNi_0.5_Mn_1.5_O_4_ full cells that exhibited excellent cycling stability, high rate capability and enhanced safety (Figure 5f–i).

Conventional Li salts e.g., LiPF_6_ and LiBF_4_ are also prepared in concentrated form to enhance their anodic stability.^33^ Concentrated electrolytes including 4.3 m (m being molality, mol kg^−1^) LiPF_6_/PC electrolyte[[qv: 33a]] and 7.25 m LiBF_4_/PC electrolyte[[qv: 33b]] display extended oxidation stability, exhibiting low irreversible capacity loss during cycling. However, both of the concentrated electrolytes do not improve cycling stability, if compared to the dilute electrolytes. This suggests that the aggregates or fluidic 3D network of anions and solvent molecules may have different structures and properties depending on the composition of concentrated electrolytes. An appropriate combination of solutes and solvents is very critical for specific electrode materials in order to alter the nature of SEI and enhance the electrochemical performances of LIBs.

### 2.2 Concentrated Electrolyte for ‘Beyond Li‐Ion’ Energy Storage Systems

#### 2.2.1 Concentrated Electrolyte for Li Metal Protection

Li metal is an ideal anode material for use in high‐energy‐density Li metal batteries because of its extremely high specific capacity (3.86 Ah g^−1^), and the lowest electrochemical potential (−3.04 V vs. standard hydrogen electrode).[[qv: 12b,34]] However, dendrite growth and limited CE during cycling have hampered its practical use in rechargeable batteries.^35^ The components of the electrolyte play a critical role in determining the cycling stability and safety of Li metal anodes.^14^, ^36^ Generally, ether solvents (e.g. DOL, DME) show better compatibility with Li metal anodes as compared to carbonates (e.g. EC, DMC, DEC), while the LiFSI salt is superior to LiTFSI and LiPF_6_ due to the formation of a stabilized SEI layer on Li metal surface. For example, in a dilute electrolyte (1 m), a dual‐salt electrolyte LiFSI‐LiTFSI/DOL‐DME outperforms the LiTFSI/DOL‐DME and is much superior over LiPF_6_/EC‐DMC in terms of improving the CE and cycling stability of Li.^37^ In particular, FSI^−^ anions competitively react with Li and lead to the formation of a much thinner and denser inorganic SEI layer enriched by LiF that was decomposed from the salt. The enhanced stability of this dual‐salt electrolyte toward Li metal anodes was ascribed to the synergistic effect of LiFSI and DOL. This suggests that the solvents also have a significant effect on the Li metal interfacial stability. In a recent publication, Miao et al. further demonstrated that by introducing 1,4‐dioxane (DX) as a co‐solvent into LiFSI/DME‐DX electrolyte, they could achieve stable cycling with high CE of ca. 98%.^38^

**Figure 6:**
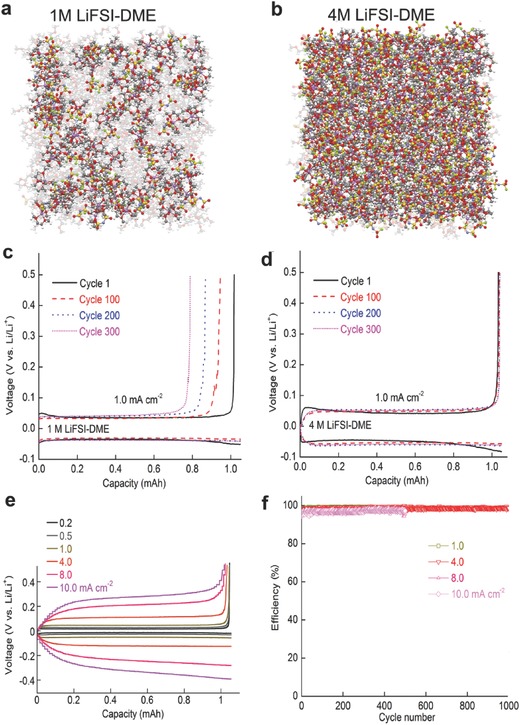
(a, b) Snapshots of the MD simulation boxes of (a) 1 m LiFSI/DME electrolyte, (b) 4 m LiFSI/DME electrolyte. Colors for different elements: Li‐purple, O‐red, N‐blue, S‐yellow, and F‐green. The uncoordinated DME solvent molecules are colored light gray. (c) Voltage profiles for the Li||Li cell cycled in 1 m LiFSI/DME; (d) Voltage profiles for the Li||Li cell cycled in 4 m LiFSI/DME; (e) Polarization of the plating/stripping for the 4 m LiFSI/DME electrolyte with different current densities. (f) CEs of Li deposition/striping in 4 m LiFSI/DME at various current densities. Reproduced with permission.^14^ Copyright 2015 Nature Publishing Group.

In addition to the chemistries of Li salts and solvents, electrolyte concentration has a significant impact on the interfacial reactions initiating on the Li metal electrode.^39^ Jeong et al. demonstrated that a concentrated electrolyte (3.27 m LiBETI/PC) produced an effective SEI on the electrodeposited Li metal in the absence of dendrite‐suppressing additives.^40^ The SEI layer formed in the concentrated electrolyte (3.27 m) is much thinner than that produced in the dilute electrolyte (1.28 m). Although the CE of ca. 80% is unsatisfactory for practical application, the results open a new direction for suppressing dendritic Li formation. Recently, Qian et al. reported that the use of highly concentrated electrolytes composed of single LiFSI salt in DME enables the high‐rate cycling of a Li metal anode with high CEs without dendrite formation.^14^ In a dilute electrolyte, uncoordinated solvent readily reacts with Li metal and leads to a low CE during cycling (**Figure** **6**a,c). In contrast, the enhanced solvent coordination with Li^+^ ions in a highly concentrated electrolyte effectively stabilized the solvent molecules and mitigated their side reactions with Li metal (Figure 6b,d). The high concentration electrolyte also facilitates the formation of a compact and highly conductive SEI layer on Li metal surface that mitigates the anion degradation during extended cycling. Using 4 m LiFSI/DME as the electrolyte, a Li||Li symmetric cell survives at 10 mA cm^–2^ for more than 6000 cycles, and a Li||Cu cell can be operated at 4 mA cm^–2^ for over 1000 cycles with an average CE of 98.4% (Figure 6d–f).

LiFSI has also been mixed with another cost‐effective Li salt for improving the CEs of Li metal and preventing dendritic Li growth. In the development of dual‐salt concentrated electrolyte, Ma et al. found that the addition of LiFSI to Lithium (fluorosulfony)(trifluoromethanesulfonyl)imide (Li(FSO_2_)N(SO_2_CF_3_), LiFTFSI) (2 m LiFSI + 1 m LiFTFSI and 2 m LiFSI + 2 m LiFTFSI in DOL/DME) could further enhance the CEs of Li metal as compared to the single‐salt electrolyte (4 m LiFTFSI).^41^ Liu et al. also systematically investigated the dual‐salt concentrated electrolyte (1 m LiFSI + 2 m LiTFSI and 2 m LiFSI + 1 m LiTFSI in DOL/DME) and found that the SEI layer on Li metal formed in these two electrolytes are more compact and thinner than that generated in the single‐salt (3 m LiTFSI) electrolyte.^42^ Consistently, both Ma et al.^41^ and Liu et al.^42^ demonstrated that the percentage of LiF content increases with the addition of LiFSI in the dual‐salt electrolytes, suggesting that the preferential decomposition of the FSI^−^ anion produces a more robust SEI film.

**Figure 7:**
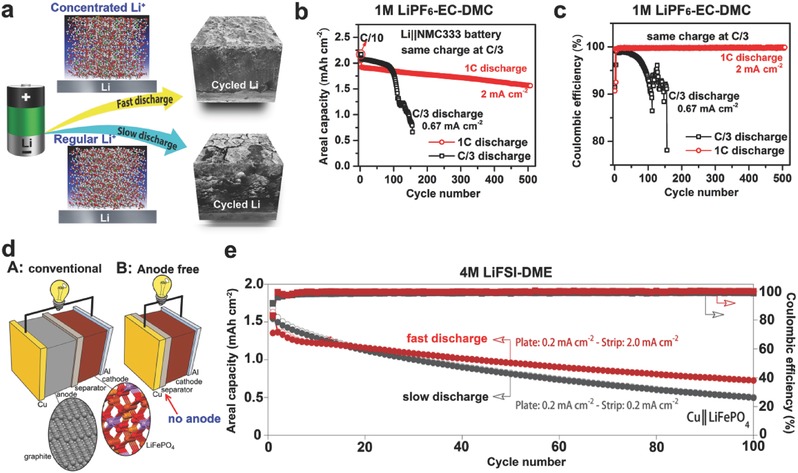
(a) Schematic illustrations of discharge C rate effect on the interfacial stability of Li metal anode. (b) Cycling stability and (c) CE of Li||NMC cells using 1 m LiPF_6_/EC‐DMC electrolyte during cycling at different discharge rages (same charge rate C/3). Reproduced with permission.^43^ (d) Schematic illustrations of battery configurations. A: Conventional LIB (Cu|C_6_||LiFePO_4_|Al). B: Anode‐free battery (Cu||LiFePO_4_|Al). (e) Capacity and CE of anode‐free Cu||LiFePO_4_ cells using 4 m LiFSI/DME charged at 0.2 mA cm^−2^ and discharged at either 0.2 or 2.0 mA cm^−2^. Reproduced with permission.^44^

Inspired by the concept of concentrated electrolyte, Zheng et al. tried to create a transient layer of highly concentrated electrolyte in the vicinity of a Li metal anode by fast Li stripping at a high discharge rate.^43^ The highly concentrated Li^+^ ions in this transient layer immediately solvate the available solvent molecules and facilitate the formation of a highly flexible and stable SEI layer on the Li metal surface, effectively mitigating the Li corrosion by free organic solvents and enabling the sustainable operation of Li metal batteries (**Figure** **7**a). Despite the use of conventional carbonate‐based electrolyte (1 m LiPF_6_/EC‐DMC), a high capacity retention >80% after 500 cycles can be accomplished for moderately high areal‐capacity (2 mAh cm^−2^) Li||NMC metal batteries at an optimized Li stripping process (2–4 mA cm^−2^) (Figure 7b,c). Recently, Qian et al. further confirmed the advantages of the fast discharge effect in their investigation of a high concentration ether based electrolyte (4 m LiFSI/DME) for anode‐free Li batteries (Figure 7d).^44^ After 100 cycles at a relatively high discharge current density (2.0 mA cm^−2^), the discharge capacity of the anode‐free Cu||LiFePO_4_ cell was ≈54% of its original value which is a great improvement relative to the cell cycled under a low discharge current density (0.2 mA cm^−2^) that retained only ≈32% of its original capacity (Figure 7e).

#### 2.2.2 Concentrated Electrolyte for Sodium (Na) Metal Protection

Recent concern about the poverty of Li sources is driving the exploration of alternative battery chemistries. Analogous to Li metal, Na metal electrodes could enable the development of relatively low cost and high‐energy‐density Na metal batteries. However, a critical challenge to employ Na metal as anode material is the high reactivity of Na metal, which is more problematic than Li metal. The reactivity of liquid non‐aqueous electrolytes with Na metal has restricted the plating/stripping CE to ≤95%, leading to the rapid capacity fading of Na metal batteries.[[qv: 4b]] As compared to Li metal batteries, electrolyte formulation optimization has been a more difficult challenge for the development of Na metal batteries. Recently, Seh et al. reported the use of an electrolyte consisting of diglyme and NaPF_6_. This electrolyte demonstrated the ability to reversibly plate and strip Na with high CE,^45^ although the full‐cell operation of Na metal batteries with Na‐ion intercalation cathodes was not reported.

**Figure 8:**
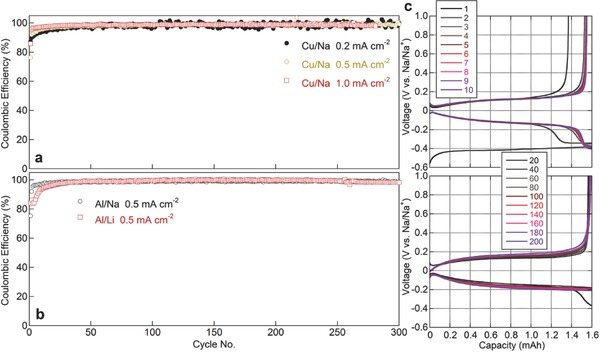
(a) CE of Na||Cu cells with 4 m NaFSI/DME electrolyte cycled at different current densities. (b) CE of Na||Al and Li||Al cells with 4 m NaFSI/DME or 4 m LiFSI/DME electrolytes, respectively, cycled at 0.5 mA cm^−2^. (c) Voltage profiles for cycling of Na||Cu cell with a 4 m NaFSI/DME electrolyte and 1.0 mA cm^−2^ current density. Reproduced with permission.^46^ Copyright 2016 Elsevier.

Similar to Li metal batteries, salt‐concentrating is an effective strategy to enhance the stability of the Na metal anode. Cao et al. demonstrated that Na metal can be reversibly plated and stripped on and off a Cu current collector with an extremely high CE (up to 99%) using concentrated electrolytes based on ether solvents (e.g., DME, diglyme, etc.) and the sodium bis(fluorosulfonyl)imide (NaN(SO_2_F)_2_ or NaFSI) salt.^46^ The 4 m NaFSI/DME electrolyte effectively discourages the parasitic reactions on Na metal during cycling because of the absence of free solvent molecules. When Cu is used as the current collector, a high CE value of 99% for the cells cycled at 1.0 mA cm^–2^ could be achieved during the long‐term cycling process (**Figure** **8**a,c). When the Cu was replaced by an Al current collector, a highly efficient cycling of Na metal was also obtained in the 4 m NaFSI/DME electrolyte (Figure 8b). The authors further demonstrated the compatibility between the concentrated NaFSI/DME electrolyte and a sodium intercalation cathode Na_3_V_2_(PO_4_)_3_. Excellent cycling performance was achieved in terms of both rate performance and the near 100% CE of the cathode, validating the concentrated NaFSI/DME electrolyte as a promising electrolyte for the develpoment of long cycle life Na metal batteries.

#### 2.2.3 Concentrated Electrolyte for Li‐S Batteries

Li–S batteries have attracted intensive attention because of their high theoretical capacity, natural abundance of elemental S, and environmental friendliness.[[qv: 2a‐c,47]] A Li‐S battery operates by the reduction of S during discharge to form soluble lithium polysulfides with different chain lengths and eventually to form insoluble Li_2_S_2_ or Li_2_S. The theoretical specific capacity and energy from Li–S batteries are 1675 Ah kg^−1^ and 2650 Wh kg^−1^ respectively, which are substantially higher than those of state‐of‐the‐art LIBs. However, fast capacity degradation and high self‐discharge rate remain significant challenges hindering the practical applications of Li–S batteries. The problems originate from the formation of a series of soluble polysulfides Li_2_S_x_ (x > 2) that could easily diffuse to the Li metal anode and participate in the notorious ‘sulfur shuttle’ reactions and corrode the Li metal anode. The former dramatically lowers the CE while the latter increases the cell impedance, shortening lifespan of Li–S batteries.^48^

Various approaches have been proposed, such as the immobilization of sulfur in a variety of hosting materials,[[qv: 2a,49]] surface modification,^50^ anode protection by employing LiNO_3_ as the electrolyte additive,^51^ and the modulation of electrolyte solution structure to address these challenges.^15^, ^52^ In the electrolyte modulation approaches, the use of concentrated electrolyte is an effective strategy to restrain the dissolution of polysulfides in the electrolyte and alleviate the ‘sulfur shuttle’ reactions, thus improving the long‐term cycle life of Li–S batteries.^15^, ^53^ Suo et al. proposed a class of solvent‐in‐salt electrolytes with LiTFSI concentrations up to 7 mol L^−1^ of DOL‐DME, which can effectively inhibit the dissolution of lithium polysulfides and also mitigate the Li metal corrosion.^15^ Electrolytes with highly concentrated Li salt have thermodynamic and kinetic benefits in manipulating the dissolution of polysulfide by the common ion effect, while the reduced free solvent molecules in the concentrated electrolyte translate to less side reactions with the Li metal anode. Zhang et al. further incorporated the viscosity effects of solvents (DME vs. tetraglyme) in different concentrated electrolytes into the observed electrochemical performance of the sulfur cathode.^54^

The concentrated anions in the electrolyte also play a critical role in dictating the interfacial properties and thus the electrochemical performances of Li–S batteries. Cao et al. performed a detailed comparison on the roles of salt anions in high concentration (3 m in DOL‐DME) electrolytes, i.e. TFSI^−^ vs. FSI^−^.^55^ It was found that the FSI^−^ anion is less stable than the TFSI^−^ anion in the Li–S battery. This is because the N–S bond in the FSI^−^ anion is considerably weak and the scission of this bond results in the formation of lithium sulfate (LiSO_x_) in the presence of polysulfide species. As a consequence, when tested at room temperature, Li–S batteries using LiFSI‐based electrolytes show much inferior cycling stability as compared to those using LiTFSI‐based electrolytes,^55^ although the LiFSI is beneficial for Li metal stability.^14^ However, Kim et al.^56^ reported that electrochemical performance of LiFSI‐based electrolyte in situ forms protecting coatings on both the cathode and anode surfaces. When tested at 60 °C, Li–S batteries using 3 m LiFSI/DME electrolyte exhibit an average CE close to 100.0% up to 1000 cycles with a capacity loss of only 24%. The controversial conclusions on LiFSI for Li‐S batteries could originate from the temperature used for battery testing i.e., 60 °C vs. room temperature (≈25 °C).^55^ At elevated temperatures, FSI^−^(–F) anion radicals generated during electrochemical reduction give rise to the formation of LiF decomposition products,^56^ which are considered to passivate the electrode surface, although how LiF passivation could suppress polysulfides dissolution is not clear. The advantage of high temperature may improve the interfacial reaction kinetics, overcoming the kinetic barrier caused by the resistive protecting surface film. In addition to the temperature, the different testing protocols e.g., S loading, binder type and amount etc., in different groups may also lead to the different effects of LiFSI on the cycling stability.^57^

**Figure 9:**
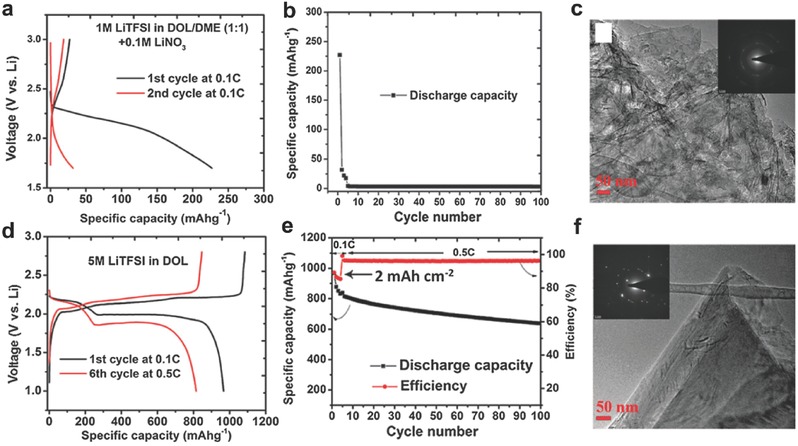
(a) Charge/discharge curves of a lithiated graphite (LG)/S full cell at 0.1C in 1 m LiTFSI/DOL‐DME with 0.1 m LiNO_3_ as an additive and (b) corresponding cycling stability and CE. (c) TEM image of the graphite after being cycled in panel (b) for 5 cycles and the corresponding electron diffraction pattern (inset). (d) Charge/discharge curves of the LG/S full cell at 0.1C and 0.5C in a 5 m LiTFSI/DOL electrolyte without LiNO_3_ and (e) the corresponding cycling performance. The areal capacity of the cathode is 2 mAh cm^−2^. (f) TEM image of the graphite after being cycled in panel (e) for 5 cycles and the corresponding electron diffraction pattern (inset). Reproduced with permission.[[qv: 60b]] Copyright 2015, Royal Society of Chemistry.

In general, the aforementioned fundamental researches about Li–S batteries are conducted by using thin film S electrodes with low S loadings of about 1 mg cm^−2^, whereas excessive Li is provided and the degradation of Li metal anode is usually ignored. However, when the S loading is raised to the practical application level (2–4 mAh cm^−2^ capacity loading), the stability of the Li metal anode then becomes the decisive factor due to its instability with the electrolyte and the continuous growth of resistive SEI on the Li metal surface. While Li anode protection remains a challenge for a long history of Li metal batteries, an alternative approach to avoid the Li metal anode degradation is to resort to the use of Si,^58^ Sn,^59^ or carbon anodes.^60^ Lu et al. proved the concept of Li‐ion sulfur batteries employing intercalation graphite compound as the anode.[[qv: 60b]] In 1 m LiTFSI in DOL‐DME, the lithiated graphite (LG)/S full‐cell shows very low initial discharge capacity and poor reversibility (**Figure** **9**a,b) due to the incompatibility between graphite and the regular EC‐free electrolyte (Figure 9c). In contrast, by increasing the concentration of LiTFSI salt to 5 m in DOL solvent, the LG/S full‐cell with sulfur loading >2 mg cm^−2^ delivers a high reversible capacity of 980 mAh g^−1^, a capacity retention of 81.3% and a high CE of above 97% after 100 cycles (Figure 9d,e). The significantly enhanced cycling performance was attributed to the enhanced interfacial stability between graphite and concentrated LiTFSI/DOL, because a very thin SEI layer was formed to protect the graphite lattice (Figure 8f). Analogously, Bhargav et al. demonstrated a high performance graphite‐polysulfide full cell using a high concentration electrolyte based on a combination of 3 m LiFSI + 1 m LiTFSI in DME.^61^ The electrolyte concentrating strategy has thus proved to be a feasible strategy for developing high performance Li‐ion sulfur batteries coupled with versatile anode materials, while Li metal issues can be completely addressed.

#### 2.2.4 Concentrated Electrolyte for Li‐O_2_ Batteries

Li–O_2_ batteries have been intensively investigated in recent years, based on their ultra‐high theoretical energy densities (3505 Wh kg^−1^).[[qv: 2b,62]] However, rechargeable Li–O_2_ batteries suffer from severe decomposition of electrolyte during discharge because the superoxide radical anions (O_2_^.−^) attack organic solvents and/or substrates.^63^ This has pushed the displacement of the carbon matrix with non‐carbon materials,^64^ and the electrolytes from carbonates to ethers,^65^ and sulfones.^66^ There is an urgent need to develop a stable electrolyte to enable the long‐term operation of rechargeable Li–O_2_ batteries.^67^ Different types of Li salt and solvents have been screened for Li–O_2_ batteries,[[qv: 63a,68]] and the effects of Li salt concentration on the electrochemical performances of Li‐O_2_ batteries have also been explored.^17^, ^69^

Li et al. demonstrated that the cycling performance of the Li–O_2_ batteries is closely related to the concentration of LiTFSI in triglyme (G3) and tetraglyme (G4).[[qv: 69b]] The molar ratio (LiTFSI:Gx) of 1:5 in both G3 and G4 offers superior cycling stability without capacity loss over 20 cycles. Li–O_2_ batteries exhibited a more stable discharge voltage in LiTFSI‐(G3)_5_ than in LiTFSI‐(G4)_5_ during cycling. They explained the dependence of cycling stability of Li–O_2_ batteries with the competitive accessibility of O_2_^.−^ between solvated Li^+^ ions and glyme molecules.[[qv: 69b]] Liu et al. further studied the nucleation and growth mechanism of Li_2_O_2_ crystals in electrolytes containing different concentrations of LiTFSI in G4.[[qv: 69c]] They demonstrated that Li^+^ ion concentration in the electrolyte tailors the Li_2_O_2_'s morphology, which is explained with two types of growth mechanisms: surface growth in dilute electrolyte and space growth at higher concentrations. At low concentration, discharge products are grown as a thin film spread on the electrode surface, impeding the charge transfer reaction to form more Li_2_O_2_ discharge product. In medium electrolyte concentrations (2–3 m), the Li_2_O_2_ discharge products tend to grow three dimensionally, enabling a high utilization of electrode volume, thus delivering the highest discharge capacity and average discharge voltage.[[qv: 69c]] At 4–5 m, oxygen transport passages could be easily obstructed by discharge pruducts, leading to lower discharge capacity. How this barrier is different with the surface passivation formed in 1 m electrolyte is not clear.

**Figure 10:**
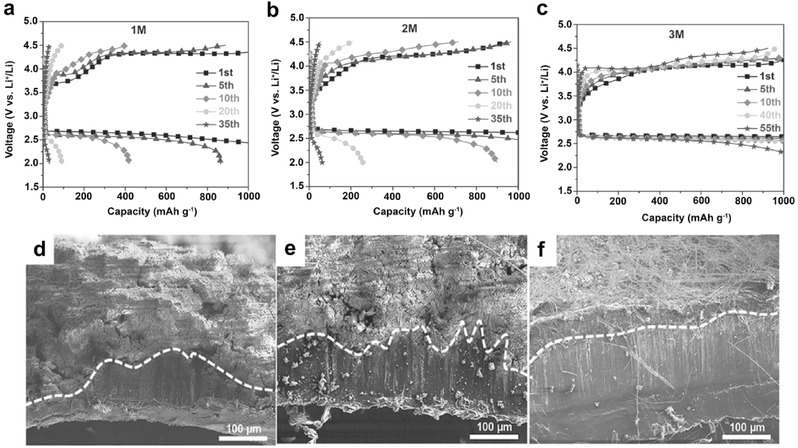
(a–c) Voltage profiles of the cells using LiTFSI/DME electrolytes with salt concentrations of a) 1 m; b) 2 m; c) 3 m. The cells were cycled using a capacity‐limited (1000 mAh g^−1^) protocol between 2.0 and 4.5 V at 0.1 mA cm^−2^. (d–f) SEM images of cross‐sectional Li‐metal anodes, after 40 cycles in LiTFSI/DME electrolytes with different salt concentrations: (d) 1 m; (e) 2 m; and (f) 3 m. Reproduced with permission.^17^

Recently, Liu et al. systematically investigated the effect of Li salt (LiTFSI) concentration in DME‐based electrolytes on the cycling stability of Li–O_2_ batteries.^17^ Cells with concentrated electrolyte demonstrated an increase in cycling stability under both full discharge/charge (2.0–4.5 V vs. Li/Li^+^) conditions and capacity‐limited (at 1000 mAh g^−1^) conditions (**Figure** **10**a–c). The improved cycling performance of Li–O_2_ batteries using 3 m LiTFSI in DME was explained with the following two aspects. On one hand, the concentrated electrolyte is more compatible with the Li metal anode due to the absence of free solvent molecules (Figure 10d–f), restraining the internal resistance increase that resulted from Li metal anode degradation.^14^ On the other hand, based on their density functional theory (DFT) calculations, in a concentrated electrolyte, all DME molecules are coordinated with salt cations. Therefore the C–H bond scission of the DME molecule became more difficult. This resulted in the decomposition of the concentrated electrolyte being thus mitigated, and both air cathodes and Li‐metal anodes exhibited much better reversibility, improving the cyclability of Li–O_2_ batteries.

Since the Li–O_2_ battery belongs to Li metal batteries, the low CE of a Li metal anode will become a serious issue for practical applications of Li–O_2_ batteries. The existence of oxygen radicals further worsens the interface side reactions on the Li metal anode. Besides LiTFSI salt, concentrated electrolytes composed of other lithium salts (e.g. LiNO_3_,^70^ LiFSI,^71^ LiTf), and solvents (e.g. DMSO, AN) are worth further studies to enable and improve long‐term cycling of Li–O_2_ batteries.

### 2.3 Concentrated Electrolyte for Aqueous Energy Storage System

The long‐existing issues for non‐aqueous battery technologies are the concerns on the safety and environmental impacts.^72^ For this reason, aqueous battery systems have regained interests in recent years for large‐scale green energy storage. However, in a conventional aqueous electrolyte, the decomposition products (H_2_, O_2_ or OH^−^) from water are incompetent to deposit in a dense solid state to function as a protective interphase (SEI layer). Therefore, the aqueous based electrolytes suffer from a narrow electrochemical stability window (ESW) (≈1.5 V), intrinsically limiting the practical operating voltage and energy (<70 Wh kg^−1^) output.^73^

Concentrated aqueous electrolyte has been discovered to expand the ESW of aqueous batteries and largely improve the cell stability during cycling. Early research has shown that a saturated LiNO_3_ aqueous solution has an ESW of about 2.8 V, far beyond that of the regular aqueous electrolyte.^74^ It was also found that the high concentration (5 m) of a LiNO_3_ based aqueous electrolyte exhibited fast electrode reaction kinetics.^75^ However, aqueous rechargeable LIBs using a saturated LiNO_3_ aqueous solution showed relatively low discharge capacity along with quick capacity fading, indicating a poor ability for LiNO_3_ to form a SEI.^74^ Similar to the case in non‐aqueous based electrolytes, Li salt has a significant effect on the interfacial reactions and the resulting SEI layer stability. Recently, Wang and Xu et al. proposed a highly concentrated aqueous electrolyte for aqueous based LIBs.^20^ The highly concentrated electrolyte contains molality >20 m LiTFSI in water, which is thus called “water‐in‐salt” (WIS) electrolyte. With a LiTFSI concentration of 21 m (cation: water ratio of 1:2.6), their molecular dynamics (MD) simulation predicted that on average two TFSI^−^ anions would be involved in each Li^+^ primary solvation sheath, leading to an interfacial chemistry dominated by the reduction of TFSI^−^ anions. This reduction process generates sufficient LiF from TFSI^−^ to form a robust anode SEI. The LiF‐rich interphase then serves as an electron barrier preventing the reduction of both TFSI^−^ anions and water molecules while still allowing for prompt Li^+^ ion conduction.

**Figure 11:**
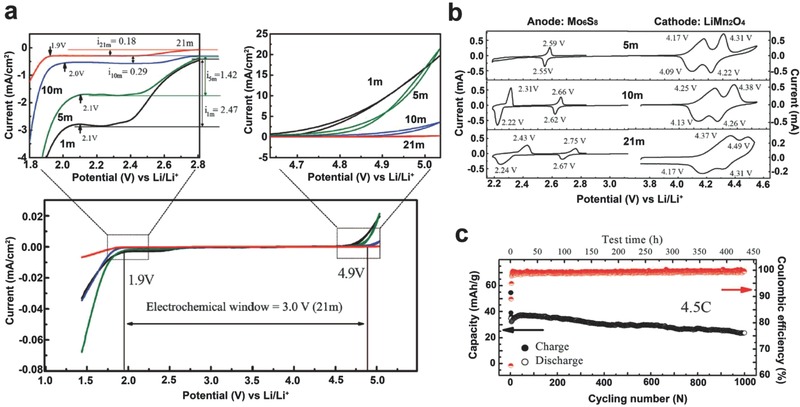
(a) The ESW of LiTFSI/H_2_O electrolytes on stainless steel working electrodes at 10 mV s^−1^. The potential was converted to Li/Li^+^ reference. (b) ESW of electrolytes with various LiTFSI concentrations as measured with CV on active (LiMn_2_O_4_ and Mo_6_S_8_) electrode surfaces at 0.1 mV s^−1^. (c) Cycling stability and CE of full aqueous Mo_6_S_8_||LiMn_2_O_4_ Li‐ion cell using 21 m LiTFSI/H_2_O electrolyte at 4.5C rate. Reproduced with permission.^20^ Copyright 2015, American Association for the Advancement of Science.

The formation of this unique electrode/electrolyte interface pushes both oxygen and hydrogen evolution potentials well beyond the stability limits of water. An ESW of ≈3.0 V (1.9–4.9 V vs. Li/Li^+^) is achieved for the concentrated solution containing 21 m LiTFSI (**Figure** **11**a). One of the most important features of the concentrated electrolyte is the shifting of the redox reaction processes toward positive potentials, thereby moving the second redox process of Mo_6_S_8_ into the expanded ESW of the WIS electrolyte (Figure 11b). Using a LiMn_2_O_4_ cathode and a Mo_6_S_8_ anode, a full LIB in WIS electrolyte cycled up to 1000 times, along with nearly 100% CE at 4.5C rate (Figure 11c).

Wang and Xu et al. further proposed a new superconcentrated aqueous electrolyte by introducing a second Li salt to the parent WIS electrolyte.^41^ The resultant superconcentrated electrolyte (28 m) consists of 21 m LiTFSI and 7 m lithium trifluoromethane sulfonate (LiOTf), also called “water in bisalts” (WIBS), and functions as a molten electrolyte with a cation/water ratio of about 1:2. This WIBS electrolyte leads to a more effective formation of a protecting interphase on the anode along with the further suppression of water activities at both the anode and cathode surfaces. The improved electrochemical stability allows for the use of TiO_2_ as the anode material. A 2.5 V aqueous LIB based on a LiMn_2_O_4_ cathode and a carbon‐coated TiO_2_ anode delivers a high energy density of 100 Wh kg^−1^, along with high CE and decent cycling stability. Because of the thermodynamic instability of the LiMn_2_O_4_ cathode, efforts have recently turned to using a LiFePO_4_ cathode.^76^ The electrochemical combination of a LiFePO_4_ cathode, a Mo_6_S_8_ anode, and WIS electrolyte led to a LIB operating at 1.3 V. This combination also exhibited a significantly improved cycling performance at high temperatures (55 °C) as well as a mitigated self‐discharge in the fully charged state. It is believed that optimizing the salt chemistry, concentration, pH value, and electrode chemistry will further push the energy densities of aqueous LIBs closer to those of the state‐of‐the‐art non‐aqueous LIBs.^77^

## 3 Mechanism

### 3.1 Solution Structure Modification

**Figure 12:**
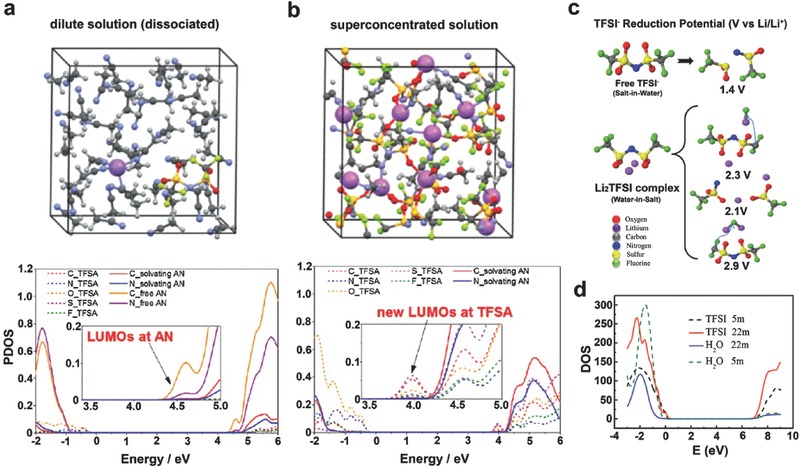
Supercells used and projected density of states (PDOS) obtained in quantum mechanical DFT‐MD simulations on non‐aqueous (a) dilute (0.4 m) and (b) superconcentrated (4.2 m) LiTFSI/AN solutions. Reproduced with permission.[[qv: 11b]] Copyright 2014 American Chemical Society. (c) Predicted reduction potential from G4MP2 quantum chemistry calculations and (d) PDOS for LiTFSI/H_2_O electrolyte from HSE06 DFT calculations. Reproduced with permission.^20^ Copyright 2015, American Association for the Advancement of Science.

As discussed above, highly concentrated Li salt in the electrolyte alters the interfacial reaction pathways and the properties of the SEI layers. The fundamental reason is usually ascribed to the significant change in the electrolyte solution structures, reduction of free solvent molecules, and the corresponding modified highest occupied molecular orbital (HOMO) and lowest unoccupied molecular orbital (LUMO) energies, according to the findings on the atypical behavior of highly concentrated non‐aqueous electrolytes.[[qv: 11b,20]] In a dilute (e.g. 1 m) LiTFSI/AN electrolyte, the stable solvation structure around Li^+^ ion is 3‐ or 4‐fold coordination. The LUMO is located on the AN molecules (**Figure** **12**a). AN molecules are predominantly reduced without forming a stable surface film, and thus Li^+^ ions cannot reversibly intercalate into the graphite electrode. As for the superconcentrated (4.2 m) electrolyte, Li^+^ ions have 2‐fold coordination on average due to the shortage of AN solvent and all the TFSI^−^ anions exist as AGGs with strong coulombic interactions with multiple Li^+^ cations (Figure 12b). Because of this coordination structure change, the conduction bands and energy levels of TFSI^−^ anions are lowered below those of AN molecules, thereby shifting LUMO to the TFSI^−^ anions. In this case, TFSI^−^ anions can be preferentially reduced to form a TFSI‐derived surface film on the graphite surface, which is considered the origin of the improved reductive stability to allow for reversible Li^+^ ion intercalation into graphite electrodes.

In a similar manner, the highly concentrated Li salts also alter the solution structure of aqueous electrolytes (Figure 12c). Xu et al. showed that in a WIS electrolyte (21 m LiTFSI in water), AGGs such as Li_2_(TFSI)(H_2_O)_x_ exhibit a reductive stability of ≈2.9 V (vs. Li/Li^+^), which is much higher than the reduction potential for isolated TFSI^−^ anions at 1.4 V (vs. Li/Li^+^) and hydrogen evolution at 2.63 V (vs. Li/Li^+^). The preferrential reduction of TFSI^−^ generates sufficient LiF to form a stabilized SEI, thereby preventing the reduction of both TFSI^−^ and water molecules, similar to a SEI layer formed in non‐aqueous electrolytes.

### 3.2 The Relation between the Electrical Double Layer and SEI

**Figure 13:**
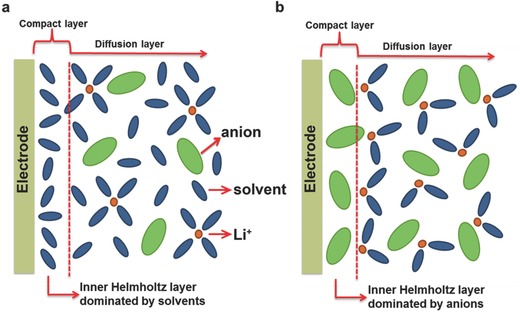
SEI layer formation mechanism in (a) dilute electrolyte and (b) concentrated electrolyte.

So far, the origin of SEI on the electrodes from the concentrated electrolytes is still unclear, but it is worth discussing the relation between the electrical double layer and the SEI first. The electrical double layer structures in dilute and concentrated electrolytes are different. In the dilute electrolyte solution, the free solvent molecules dominate the inner Helmholtz layer (**Figure** **13**a), which dictates the side reactions on the electrode once the electrical field is applied. The SEI layer is therefore mainly derived from the decomposition of solvents, which is consistent with the conventional wisdom on SEI formation. However, in the concentrated electrolyte, the high concentration of Li salt increases the association between Li^+^ ions and the solvent molecules, reducing the presence of the free solvent molecules. Not only do the anions of Li salt enter the Li^+^ ion solvation structure, they also move into the inner Helmholtz layer (Figure 13b). Therefore, when the electrode is polarized, more anions get decomposed, strengthening the anion contribution to the SEI components. Consequently, in concentrated conditions, anions in the inner Helmholtz layer play a more important role in modifying the properties of the SEI layer formed.

**Figure 14:**
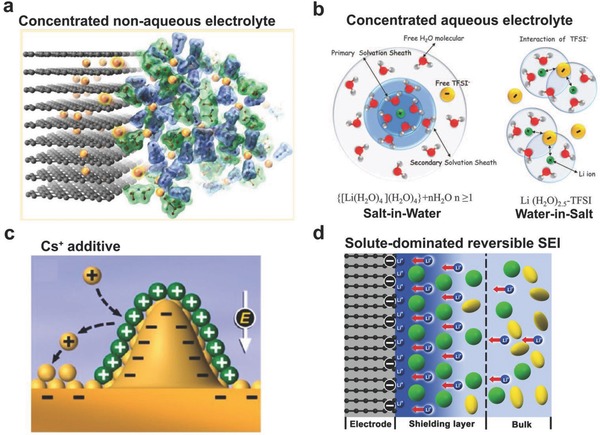
(a) Improved interfacial stability in non‐aqueous (AN‐based) superconcentrated electrolyte. Reproduced with permission.[[qv: 11b]] Copyright 2014 American Chemical Society. (b) Illustration of the Li^+^ ion primary solvation sheath in diluted and water‐in‐salt solutions. Reproduced with permission.^20^ Copyright 2015, American Association for the Advancement of Science. (c) Illustration of Li dendrite prevention mechanism by using large cations (Cs^+^) additive. Reproduced with permission.^78^ Copyright 2013 American Chemical Society. (d) Illustration of the formation mechanism of solute‐dominated reversible SEI. Reproduced with permission.^80^ Copyright 2017 American Chemical Society.

In this regard, all the electrolyte concentrating strategies could, in fact, be considered to deplete the solvent molecules in the inner Helmholtz layer by reducing the number of solvent molecules and increasing the salt anions at the same time. Such change of the inner Helmholtz layer greatly alters the SEI layer formation process. The SEI layer formed in concentrated electrolytes, typically rich in LiF, is thinner and more compact, effectively suppressing the further reactions between active electrode and the electrolyte. This functioning mechanism not only fits for non‐aqueous electrolytes for Li based batteries (**Figure** **14**a),[[qv: 11b]] but also could be widely applied to other energy storage systems using aqueous based concentrated electrolytes (Figure 14b),^20^ including Li^+^ ion based aqueous batteries as well as Na^+^ ion based aqueous batteries.

When the electrical double layer is correlated to the origin of SEI layers, many other reported strategies can be considered as tuning the Helmholtz layer, which then affects the SEI formation process. For example, the solvation ability of large cations, such as Cs^+^, is quite different with Li^+^; the alteration of the electrical double layer after incorporating Cs^+^ may reduce the available solvent molecules in the vicinity of the Li metal anode. In addition to the self‐shielding mechanism proposed by the authors, the original SEI evolved from the double layer should also be considered. A very different Li deposition process has been discovered partially due to the component change of the initial SEI (Figure 14c).^78^

### 3.3 Reversible SEI Formation

A very critical factor that has been ignored in all the aforementioned work is the influence from the electrical field on the solubility of the concentrated electrolyte. It is well known in solution chemistry that the electric field largely accelerates the nucleation/precipitation process of crystals in solutions,^79^ which is, unfortunately, not considered in literature.

**Figure 15:**
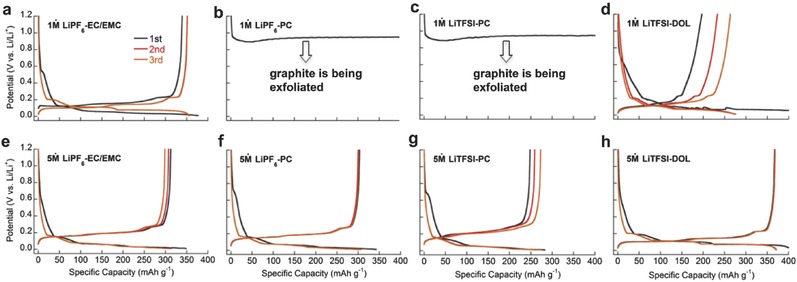
Lithiation/delithiation (1st, 2nd and 3rd) profiles of graphite in Li||graphite half cells (25°C and 0.1C) with different electrolyte solutions. (a) 1 m LiPF_6_/EC‐EMC. (b) 1 m LiPF_6_/PC. (c) 1 m LiTFSI/PC. (d) 1 m LiTFSI/DOL. (e) 5 m LiPF_6_/EC‐EMC. (f) 5 m LiPF_6_/PC. (g) 5 m LiTFSI/PC. (h) 5 m LiTFSI/DOL. The “concentration” in each electrolyte is calculated by using the mole of salt divided by the volume of solvent used to dissolve the salt. Reproduced with permission.^80^ Copyright 2017 American Chemical Society.

**Figure 16:**
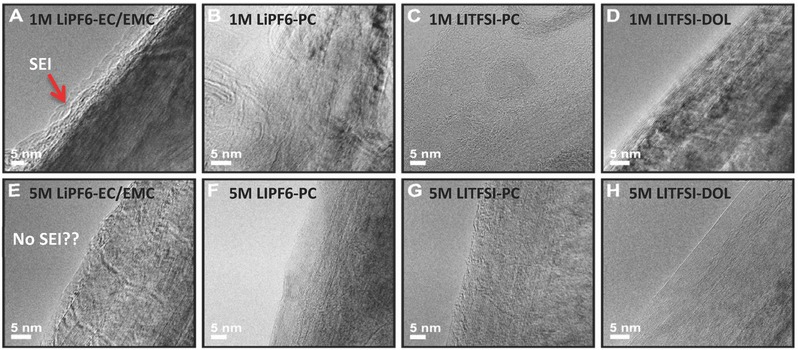
HR‐TEM images of the graphite electrodes after cycling in (A) 1 m LiPF_6_/EC‐EMC. (B) 1 m LiPF_6_/PC. (C) 1 m LiTFSI/PC. (D) 1 m LiTFSI/DOL. (E) 5 m LiPF_6_/EC‐EMC. (F) 5 m LiPF_6_/PC. (G) 5 m LiTFSI/PC. (H) 5 m LiTFSI/DOL. Graphite are completely exfoliated in (B) and (C). Reproduced with permission.^80^ Copyright 2017 American Chemical Society.

More recently, Lu and Xiao et al. proposed a reversible SEI formation mechanism that emphasizes the precipitation of solute from concentrated solutions under an electrical field. (Figure 14d).^80^ They found that a wide range of concentrated electrolytes, regardless of their specific compositions, could enable the reversible cycling of graphite without EC (see **Figure** **15**). No SEI layers were found on the graphite electrodes in contact with concentrated electrolytes (**Figure** **16**). It is hypothesized that a reversible protecting layer, precipitated from partially solvated salt, can be induced by an electric field on the electrode surface in contact with a highly concentrated electrolyte—regardless of its specific composition (see Figure 14d for the hypothesis). Once the electrode is unpolarized, the surface reverts to its original structure which is essentially “reversible”. Traditional SEI components decomposed from solvents should still exist but are not dominant any longer in concentrated electrolytes.

## 4 Conclusions and Perspectives

Concentrated electrolytes are attracting increasing amounts of interest due to the unique SEI properties identified recently in both non‐aqueous and aqueous types. Currently, the formation mechanisms and the constituents of the SEI derived from concentrated electrolytes can be categorized into three groups: 1) the considerably decreased irreversible solvent reduction due to the significantly reduced number and activity of free solvent molecules, 2) the thin and robust inorganic SEI film typically rich in LiF derived from the sacrificial anion reduction which significantly enhances the interfacial stability of various energy storage systems, and 3) the reversible SEI formation from the precipitation of the solute induced by the electrical field. As more research efforts are dedicated in this field, the understanding of the fundamental mechanisms in this intriguing system will be further deepened. MD and DFT simulations will be a complementary approach in assisting the massive screening of suitable concentrated electrolytes for various applications. Modeling the interface, although very challenging, may need more attention to promote further understanding of the SEI formation in concentrated electrolytes and on different electrode surfaces.

Meanwhile, some general drawbacks from adopting concentrated electrolytes should also be kept in mind. For example, the concentrated Li salts may precipiate at low temperatures which will largely affect the safety and performances of cells. Some of the concentrated electrolytes may be very difficult to wet cell separators and the thick electrodes required to meet the high energy goals of the batteries. The cost of concentrated electrolyte should be considered since Li salt is more expensive than solvents. Future work needs to remove the aforementioned hurdles and identify approaches to create the same unique SEI functionalities between eletrodes and electrolytes without having to significantly increase the electrolyte concentration. Dilution of concentrated electrolyte with other inert solvents, which does not solvate with Li^+^ ions but has wide electrochemical stability range, would be one of the most facile strategies to widen the operating temperature range, improve the wetting ability, and lower the cost.^81^ It may also be possible to tune the eletrical double layer through certain additives that prefer to adsorb on electrode surfaces and thus modify the SEI constituents, which may be more adaptable by industry. Combining concentrated electrolytes with solid state electrolytes may also uncover new findings in the future to accelerate the market penetration of high energy and safe batteries for a wide range of applications.
